# Cardiac Health Assessment Using a Wearable Device Before and After Transcatheter Aortic Valve Implantation: Prospective Study

**DOI:** 10.2196/53964

**Published:** 2024-06-03

**Authors:** Rob Eerdekens, Jo Zelis, Herman ter Horst, Caia Crooijmans, Marcel van 't Veer, Danielle Keulards, Marcus Kelm, Gareth Archer, Titus Kuehne, Guus Brueren, Inge Wijnbergen, Nils Johnson, Pim Tonino

**Affiliations:** 1 Department of Cardiology, Catharina Hospital Eindhoven, Eindhoven, Netherlands; 2 Philips Research Department, Eindhoven, Netherlands; 3 Department of Cardiology, RadboudUMC, Nijmegen, Netherlands; 4 Department of Biomedical Engineering, Eindhoven University of Technology, Eindhoven, Netherlands; 5 Deutsches Herzzentrum der Charité, Institute of Computer-assisted Cardiovascular Medicine, Berlin, Germany; 6 Department of Cardiology, Sheffield Teaching Hospital, Sheffield, United Kingdom; 7 Weatherhead PET Imaging Center for Preventing and Reversing Atherosclerosis, Houston, TX, United States; 8 Division of Cardiology, Department of Medicine, McGovern Medical School at UTHealth, Houston, TX, United States

**Keywords:** aortic valve stenosis, health watch, quality of life, heart, cardiology, cardiac, aortic, valve, stenosis, watch, smartwatch, wearables, 6MWT, walking, test, QoL, WHOQOL-BREF, 6-minute walking test

## Abstract

**Background:**

Due to aging of the population, the prevalence of aortic valve stenosis will increase drastically in upcoming years. Consequently, transcatheter aortic valve implantation (TAVI) procedures will also expand worldwide. Optimal selection of patients who benefit with improved symptoms and prognoses is key, since TAVI is not without its risks. Currently, we are not able to adequately predict functional outcomes after TAVI. Quality of life measurement tools and traditional functional assessment tests do not always agree and can depend on factors unrelated to heart disease. Activity tracking using wearable devices might provide a more comprehensive assessment.

**Objective:**

This study aimed to identify objective parameters (eg, change in heart rate) associated with improvement after TAVI for severe aortic stenosis from a wearable device.

**Methods:**

In total, 100 patients undergoing routine TAVI wore a Philips Health Watch device for 1 week before and after the procedure. Watch data were analyzed offline—before TAVI for 97 patients and after TAVI for 75 patients.

**Results:**

Parameters such as the total number of steps and activity time did not change, in contrast to improvements in the 6-minute walking test (6MWT) and physical limitation domain of the transformed WHOQOL-BREF questionnaire.

**Conclusions:**

These findings, in an older TAVI population, show that watch-based parameters, such as the number of steps, do not change after TAVI, unlike traditional 6MWT and QoL assessments. Basic wearable device parameters might be less appropriate for measuring treatment effects from TAVI.

## Introduction

As transcatheter aortic valve implantation (TAVI) for severe aortic stenosis is increasingly used for older adults, including a high percentage of patients with substantial comorbidity, improvement in quality of life (QoL) is as important as extending life expectancy [1,2]. Not all TAVI patients benefit from improved physical activity, as assessed by a 6-minute walking test (6MWT) or the QoL questionnaire [3,4], nor does physical activity change the incidence of aortic stenosis [5]. Nevertheless, improvement in, for example, the baseline 6MWT distance in TAVI studies can be a marker for better survival [6,7]. However, these tests could be influenced by other factors and comorbidities such as peripheral vascular disease for the 6MWT or depression for the QoL questionnaire. Another concern with such tools is that they merely provide a snapshot of a patient’s life and might change under different circumstances. Consequently, an unbiased and longer-term tool to anticipate the benefit from TAVI would allow physicians and patients to personalize treatment and expectations.

In recent years, digital health has begun to transform medicine [8]. Smart phones and health watches, in particular, have found their way into the clinic [9]. These devices can detect atrial fibrillation [10], predict the wearer’s 5-year risk of dying [11], and aid in primary prevention to reduce the risk of atherosclerotic cardiovascular disease [12]. The wearable device used in this study, the Philips Health Watch [13], continuously measures physical parameters such as heart rate (HR), number of steps, and amount of physical activity. Combining parameters from the health watch might facilitate a more physiological and comprehensive assessment of functional status before and after TAVI. After intervention for aortic stenosis, patient symptoms often improve (as measured using a QoL questionnaire), but the question arises whether they objectively become more active as measured using a wearable tracker (cq, do patients become more active after TAVI or do they exhibit the same daily routine as that prior to TAVI?). Despite advancements in digital health, current controlled clinical assessments often rely on controlled tests such as the 6MWT and a QoL questionnaire, which may not fully capture the nuances of patients’ daily lives.

In this study, we evaluated the change in parameters collected using the Philips Health Watch (“daily activity parameters” such as walking distance) among patients before and after TAVI in comparison to standard clinical and research tests (“controlled environment tests” such as the 6MWT and QoL questionnaire). We hypothesized that after a TAVI procedure, physiological parameters such as step count, total activity time, and daily total energy expenditure (TEE) would increase, whereas respiration rate and HR would decrease.

## Methods

This prospective exploratory study sought to identify parameters from the Philips Health Watch (DL8791, Philips) that changed after successful TAVI, and their relationship with standard clinical and research tests (including the 6MWT and QoL questionnaire).

### Ethical Considerations

The study complied with the tenets of the Declaration of Helsinki and local regulations. All participants provided written informed consent, and this study was approved by an independent medical ethics committee (MEC-U approval ID: W16.141).

### Study Population

Between July 2017 and September 2018, a total of 100 consecutive patients (aged ≥18 years) with severe aortic valve stenosis undergoing a clinically indicated TAVI after the Heart Team’s decision were included. Exclusion criteria were immobility and not being able to wear an electronic health watch. All patients were recruited at the Catharina Hospital in Eindhoven, the Netherlands.

### Study Protocol

Before TAVI, all patients underwent transthoracic echocardiography, computed tomography for valve sizing and access site evaluation, laboratory testing, and clinical assessment per local protocol. Patients were screened at the outpatient clinic, and eligible and consenting patients received the Philips Health Watch. The watch was placed around the patient’s wrist after configuration with patient-specific parameters (height, weight, resting HR, and birth year). It was locked on the time screen, thereby blinding patients from all activity parameters, and worn for a week before being returned for data extraction. TAVI took place within 3-6 months of the baseline assessment. Three months after the TAVI procedure, patients visited the outpatient clinic for follow-up and again wore the Philips Health Watch for 1 week. At baseline and follow-up, a 6MWT and questionnaire (transformed WHOQOL-BREF) were administered [14].

### Analysis of the Health Watch Data

**Figure 1.**
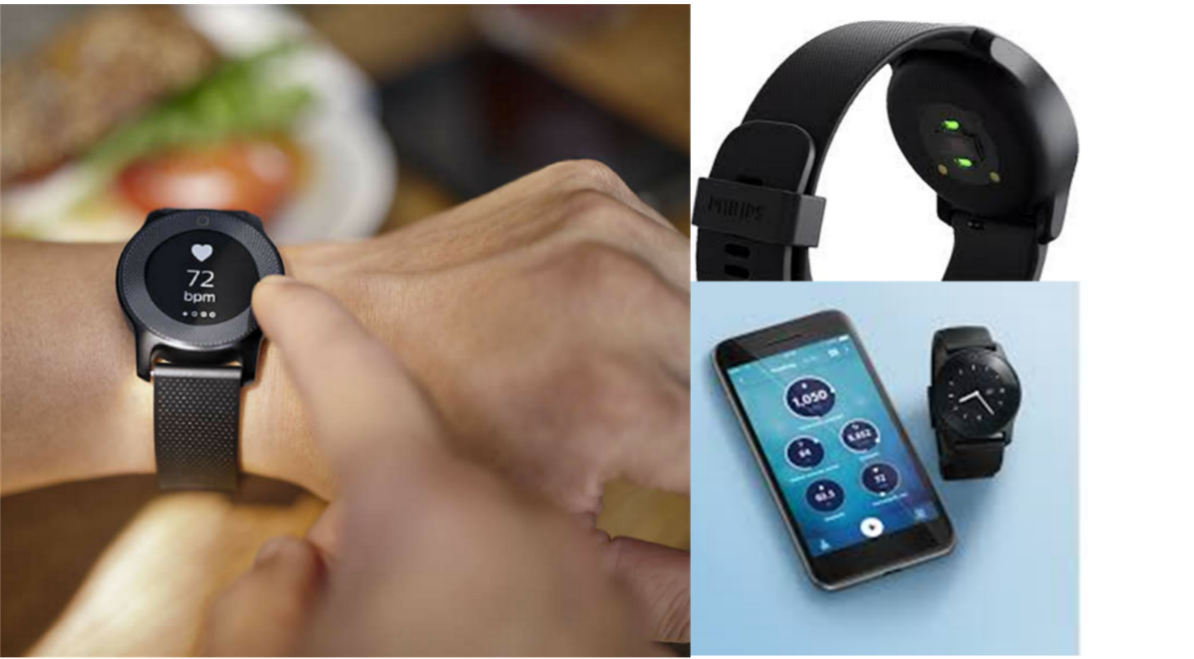
The Philips Health Watch. The Philips Health Watch is a wrist-worn, photoplethysmography-based, heart rate and activity monitor. It measures parameters such as heart rate, respiratory rate, step count, total energy expenditure, and activity time. Measurements use a 1-Hz sampling rate and are stored on the device as 1-minute average values. Data can be extracted in the hospital via Bluetooth using a proprietary iOS application and sent through Wi-Fi to the Philips Research server for analysis.

The Philips Health Watch is a wrist-worn, photoplethysmography-based, HR and activity monitor (Figure 1). Once per minute, it measures parameters such as HR, respiration rate, step count, and TEE (ie, the number of calories needed to carry out physiological functions such as breathing and physical activity, but excluding the energy required for digesting food) as described previously by Hendrikx et al [13]. Parameters are measured at a 1-Hz sampling rate and stored on the device as 1-minute average values. Data can be extracted via Bluetooth by means of an iPod, using a proprietary iOS application from Philips, and sent to a Philips Research server for use in analyses.

**Figure 2.**
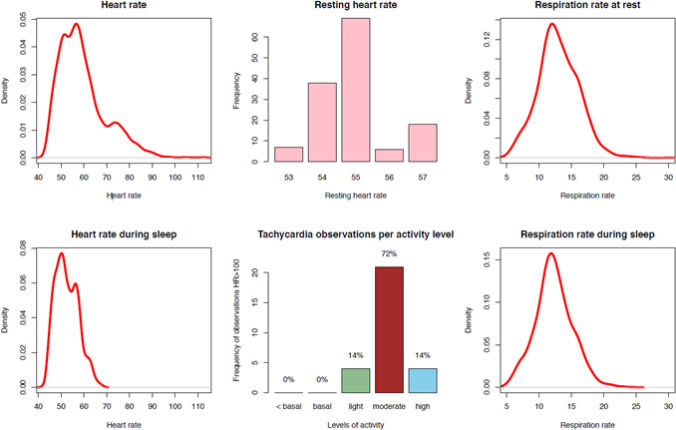
Distributions of heart and respiratory rate (overall percentage of heart rate observations <60: 57.9%; overall percentage of heart rate observations >100: 0.3%). This page of the output report from the health watch depicts density plots of the heart and respiration rates during the day and during sleep. It also shows the frequency of resting heart rate and the distribution of activity levels for heart rate observations >100 beats per minute.

**Figure 3.**
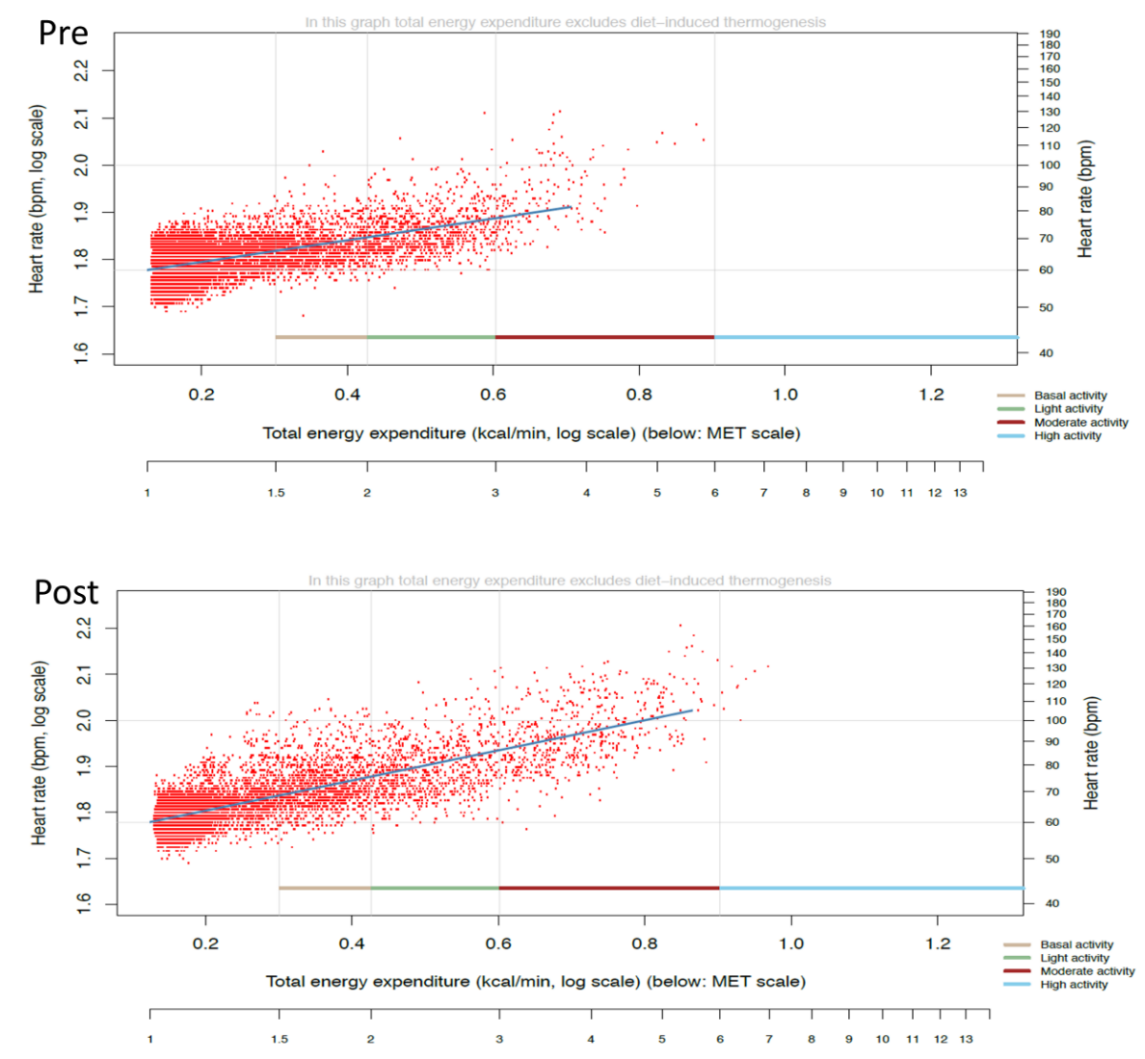
Log plot of heart rate versus total energy expenditure. Another page of the report plots each heart rate and corresponding total energy expenditure on a log plot, divided into subcategories of the Metabolic Equivalent Task (MET) scale. The fitted line relates to the energy efficiency of the cardiovascular system. bpm: beats per minute.

A full report including primary data from the watch and derived parameters consists of a summary averaged over 1 day (Table 1), distributions of HR and respiration rate (Figure 2), and log plot of the HR and TEE (Figure 3). TEE is divided into subcategories of the Metabolic Equivalent Task (MET) scale. As the older TAVI population of this cohort seemed rather inactive, a subdivision of the MET scale was designed: basal activity corresponded to a MET score of 1.5 to 2, light activity from 2 to 3, moderate activity from 3 to 6, and high activity from 6 upwards (we used standard thresholds for the last 2 categories) [15].

Each red dot in Figure 3 represents a particular measurement: the 1-minute average HR and corresponding energy expenditure level. The fitted line quantifies the cardiac energy expenditure slope (CEES): as the HR increases, more energy is needed to maintain the resulting hemodynamic state. When the slope is less steep, more energy is needed to maintain an HR of, for example, 60 beats per minute. Conversely, when the slope is steeper, less energy is needed to maintain the same hemodynamic state. The steepness of the CEES potentially serves as an indicator for the energy efficiency of the cardiovascular system.

The report and concept of CEES were proposed and made available as data derived from the raw data from the health watch by HtH from Philips Research Eindhoven and used in clinical data analysis at Catharina Hospital in Eindhoven.

**Table 1.** Watch data before and after transcatheter aortic valve implantation (TAVI; overall and good responders cohorts).

Parameter	Before TAVI (n=97)	After TAVI (n=75)	*P* value	Good responders before TAVI (n=43)	*P* value^a^
Resting heart rate (1/minute), mean (SD)	62.5 (8.9)	62.3 (8.2)	.88	62.6 (10.3)	.90
Respiratory rate at rest (1/minute), mean (SD)	16.5 (1.9)	16.2 (1.8)	.54	16.5 (2.0)	.70
Heart rate (1/minute), mean (SD)	69.9 (8.3)	69.5 (7.3)	.72	70.2 (9.6)	.95
Heart rate during sleep (1/minute), mean (SD)	63.9 (8.9)	63.3 (8.5)	.57	64.3 (10.0)	.61
Respiratory rate during sleep (1/minute), mean (SD)	16.1 (2.1)	15.9 (2.1)	.92	16.1 (2.3)	.67
Daily percentage of HR^b^ observations <60: bradycardia, median (IQR)	10.1 (1.2-34.5)	14.7 (2.4-35.4)	.92	13.3 (0.8-3.0)	.46
Daily percentage of HR observations >100: tachycardia, median (IQR)	1.3 (0.3-2.9)	1.3 (0.5-2.2)	.96	1.0 (0.3-1.0)	.86
Daily total number of steps, median (IQR)	3586 (2607-4946)	4341 (2093-6083)	.36	3633 (2763-5135)	.18
Daily cumulative active energy expenditure (kcal), mean (SD)	718.5 (206.5)	722.6 (226.6)	.98	733.0 (221.4)	.15
Daily cumulative total energy expenditure (kcal), mean (SD)	2313.3 (401.3)	2296.1 (436.6)	.72	2310.7 (428.1)	.19
Slope of log(HR/TEE^c^)	0.27 (0.21-0.34)^d^	0.29 (0.23-0.36)^d^	.26	0.26 (0.1)^e^	.04
Daily sleep time (hours)	7.9 (1.8)^e^	10.1 (15.7)^e^	.23	8.2 (7.0-8.7)^d^	.28
Daily basal activity time (minutes), median (IQR)	209 (173-253)	198 (167-262)	.43	209 (173-261)	.94
Daily light activity time (minutes), mean (SD)	183.1 (83.8)	190.3 (100.3)	.63	195.2 (88.8)	.63
Daily moderate activity time (minutes)	48.8 (60.7)^e^	58.3 (51.3)^e^	.10	20.2 (8.8-66.3)^d^	.01
Daily high activity time (minutes), median (IQR)	0.0 (0.0-0.0)	0.0 (0.0-1.5)	.18	0.0 (0.0-0.0)	.19
Daily total active (minutes), mean (SD)	101.9 (91.4)	102.3 (71.9)	.98	108.3 (100.3)	.41

a *P* values apply to the comparison between pre- and post-TAVI values in the good responders cohort. See Table S3 in Multimedia Appendix 1 for the full comparison.

b HR: heart rate.

c TEE: total energy expenditure.

d Median (IQR) values.

e Mean (SD) values.

### Statistical Analysis

Analyses were performed using SPSS (version 29.0; IBM Corp). The data are displayed as mean and SD values unless stated otherwise. Dichotomous variables are displayed as percentages (%) and absolute numbers (n). Applicable tests were 2-tailed, and *P*<.05 was considered statistically significant. Student *t* tests were used to compare normally distributed variables. Categorical variables were analyzed using the chi-square test, Fisher exact test, or McNemar-Bowker test, whichever was appropriate. As this was an exploratory study, no sample size was prespecified. Analyses were performed for the overall population (“overall cohort”), men versus women (“gender cohort”), those older than and those younger than 81 years (“81 y cohort”), those older than and those younger than 85 years (“85 y cohort”), and a cohort that had an increase of >40 m on the 6MWT after TAVI (“good responders cohort”). The 81-year cutoff is based on the median age, the 85-year cutoff was arbitrary, and the good responders cutoff is based on data from Tuttle et al [16].

## Results

### Demographic Characteristics

A total of 100 participants were enrolled. Their demographics and medications (before and after TAVI) are displayed in Table 2 and Table 3, respectively. After TAVI, 11 patients died and 11 patients were lost to follow-up. Data extraction for 3 patients failed before TAVI. Complete watch data were thus obtained for 97 patients before TAVI and 75 patients post TAVI. The population consisted of more men (n=57, 57%) than women. Demographic characteristics were representative of a clinical TAVI population with a median age of 81.0 years, NYHA (New York Heart Association) class II or higher in 92% (n=92) of patients, hypertension in 66% (n=66) of patients, and dyslipidemia in two-thirds of patients (n=67, 67%). All patients fulfilled the criteria for severe aortic stenosis. Procedural data can be found in the supplemental material in Multimedia Appendix 1.

**Table 2.** Demographic characteristics at baseline.

Characteristics			Overall cohort (N=100)	Pre-TAVI^a^ good responders cohort (n=43)	*P* value^b^
Age (years), median (IQR)			81.0 (76.0-84.0)	81.0 (74.0-84.0)	N/A^c^
Male participants, n (%)			57 (57)	22 (51)	N/A
**Risk factors, n (%)**					
	Active smoking		8 (8)	5 (12)	.19
	Hypertension		66 (66)	31 (72)	.15
	Dyslipidemia		67 (67)	28 (65)	.36
	Diabetes mellitus		23 (23)	6 (14)	.13
**Cardiac history, n (%)**					
	Prior myocardial infarction		31 (31)	14 (33)	.86
	Prior PCI^d^		41 (41)	21 (49)	.33
	Prior CABG^e^		22 (22)	12 (28)	.55
**Cardiovascular disease, n (%)**					
	Cerebral vascular disease		18 (18)	6 (14)	.37
	Peripheral vascular disease		16 (16)	5 (12)	.74
	COPD^f^		27 (27)	13 (30)	.42
	Permanent pacemaker		9 (9)	4 (9)	>.99
**Laboratory values**					
	Hemoglobin (mmol/L), mean (SD)		8.0 (0.9)	7.9 (0.7)	.42
	hs-cTnT^g^ (ng/L) level, median (IQR)		21.0 (14.0-37.8)	23.5 (15.0-34.7)	.63
	NT-proBNP^h^ (pmol/L), median (IQR)		1484 (835-3178)	1250 (835-2763)	.79
	Creatinine (mg/dL), median (IQR)		97.0 (77.0-119.0)	101.0 (81.0-118.0)	.93
**Echocardiographic parameters**					
	Left ventricular ejection fraction (%), median (IQR)		56 (46-63)	56 (42-64)	.62
	AV^i^ maximum velocity (cm/second), median (IQR)		424 (381-467)	412 (386-464)	.96
	AV mean pressure gradient (mm Hg), mean (SD)		45 (14)	42 (13)	.97
	AVA^j^ (cm^2^), mean (SD)		0.8 (0.2)	0.7 (0.1)	.70
**Symptoms, n (%)**					
	**NYHA^k^** **heart failure class**				.15
		I	8 (8)	4 (9)	
		II	26 (26)	6 (14)	
		III	57 (57)	21 (63)	
		IV	6 (6)	5 (12)	
	CCS^l^ angina grade≥III		21 (21)	9 (21)	.41
	Syncope		9 (9)	3 (7)	.42

a TAVI: transcatheter aortic valve implantation.

b *P* values correspond to the comparison between the good responder cohort versus non–good responders before TAVI.

c N/A: not applicable.

d PCI: percutaneous coronary intervention.

e CABG: coronary artery bypass grafting.

f COPD: chronic obstructive pulmonary disease.

g hs-cTnT: high-sensitivity cardiac troponin T.

h NT-proBNP: N-terminal B-type natriuretic peptide.

i AV: aortic valve.

j AVA: aortic valve area.

k NYHA: New York Heart Association.

l CCS: Canadian Cardiovascular Society.

**Table 3.** Medications.

	Before TAVI^a^, n (%)	Post TAVI, n (%)	*P* value
Aspirin	45 (56)	45 (56)	.21
Antiplatelet	28 (35)	72 (90)	<.001^b^
Anticoagulation	38 (47)	34 (43)	.77
Beta-blocker	56 (70)	38 (48)	.26
RAAS^c^	58 (73)	40 (50)	.21
Potassium sparing diuretic	12 (15)	8 (10)	>.99
Statin	61 (76)	41 (51)	.07
Calcium channel blocker	23 (29)	15 (19)	>.99
Nitrates	22 (28)	14 (18)	.15
Alpha blocker	10 (13)	7 (9)	>.99
Antiarrhythmic	5 (6)	5 (6)	>.99
Insulin	12 (15)	7 (9)	.13
Oral diabetic	23 (29)	17 (21)	.63
Loop diuretic	38 (48)	30 (38)	.75

a TAVI: transcatheter aortic valve implantation.

b *P*<.05.

c RAAS: renin-angiotensin-aldosterone system.

### Health Watch Parameters

Changes in all health watch parameters before and after TAVI are displayed in Table 1 and Table S3 in Multimedia Appendix 1 for the good responders cohort. Notably, in the total cohort, no parameter changed significantly. For the female cohort, there was a small increase in the light-to-moderate activity time (206.6 minutes before vs 207.3 minutes after TAVI, *P*=.03). For responders younger than 81 years in the good responders cohort, there was an increase in daily moderate activity: 14.2 minutes versus 39.3 minutes (*P*=.02) and 20.2 minutes versus 71.5 minutes (*P*=.01), respectively. A slight decrease was seen in the respiratory rate for responders older than 81 years (16.1 vs 15.1 per minute, *P*=.04). Heart rate, total number of steps, and daily total active minutes did not change after TAVI compared to those before TAVI for the overall group or for the different subgroups. There was no decrease in HR after TAVI despite a trend toward decreased use of beta-blockers (70% before TAVI vs 48% post TAVI, *P*=.26). Univariate analysis of health watch data could not identify a predictor for the good responders cohort (a >40 m improvement during the 6MWT). The results are displayed in Table S2 in Multimedia Appendix 1.

### Energy Efficiency of the Cardiovascular System

The CEES—the slope of the fitted line in Figure 3 between HR and TEE on a log scale—serves as an indicator for the energy efficiency of the cardiovascular system. The CEES did not change significantly before versus after TAVI for the overall cohort (*P*=.26) but increased significantly for the good responders cohort (*P*=.04).

### 6MWT and the Transformed WHOQOL-BREF Questionnaire

The distance on the 6MWT increased after TAVI compared to that before TAVI (342.8 vs 289.7 m, respectively; *P*<.001) both for the total cohort as well as all subgroups. An improvement in the physical limitation score (domain 1 of the questionnaire) could be seen in the overall cohort (from 54.5 to 61.4, *P*=.005); on subgroup analyses, male participants (55.4 vs 62.0, *P*=.03), those younger than 81 years (51.8 vs 61.1, *P*=.001), and those younger than 85 years (54.4 vs 60.8, *P*=.01) showed a similar improvement. Results from the other domains (psychological, level of independence, social relationships, and overall) did not change for the total cohort. However, we detected an improvement in the psychological domain score among participants older than 81 years (67.6 vs 71.7, *P*=.03) and in the overall score among participants younger than 81 years (248.4 vs 267.1, *P*=.009). The parameters in the 6MWT and QoL questionnaire are summarized in Table 4 and Table 5, respectively.

**Table 4.** Parameters in the 6-minute walking test (6MWT) before and after TAVI^a^.

Parameter	Before TAVI (N=100)	Post TAVI (n=76)	*P* value
Heart rate before the 6MWT (beats per minute), median (IQR)	69 (62 to 79)	70 (61 to 75)	.66
SpO_2_^b^ before the 6MWT (%), median (IQR)	97 (96 to 98)	98 (97 to 99)	.005
Distance (m), mean (SD)	289.7 (123.0)	342.8 (121.6)	<.001
Heart rate post the 6MWT (beats per minute), median (IQR)	88 (77 to 103)	89 (79 to 98)	.88
SpO_2_ after the 6MWT (%), median (IQR)	97 (95 to 98)	97 (94 to 98)	.17
Beats Above Baseline Index, median (IQR)	19 (10 to 28)	20 (12 to 29)	.99
SpO_2_ difference (%), IQR	–1 to 1	N/A^c^ to 3	.09
Walking speed (m/second), mean (SD)	0.8 (0.3)	1.0 (0.3)	<.001

a TAVI: transcatheter aortic valve implantation.

b SpO_2_: peripheral pulse oximeter saturation.

c N/A: not applicable.

**Table 5.** Quality of life questionnaire before versus post TAVI^a^.

	Before TAVI (N=100)	Post TAVI (n=76)	*P* value
Domain I - physical, mean (SD)	54.5 (18.7)	61.4 (19.7)	.005
Domain II - psychological, mean (SD)	68.1 (14.7)	67.4 (14.4)	.64
Domain III - level of independence, median (IQR)	69 (53-75)	69 (56-75)	.88
Domain IV - social relationships, mean (SD)	71.4 (15.2)	73.5 (15.8)	.31
Overall score, mean (SD)	258.8 (53.6)	268.7 (58.7)	.16

a TAVI: transcatheter aortic valve implantation.

## Discussion

### Principal Findings

To our knowledge, this is the first study in which extensive 1-week physiological data before and after TAVI were assessed using a sophisticated wearable sensor, the Philips Health Watch. Watch parameters such as activity time and step count did not increase after TAVI for the overall cohort, in contrast with significant improvements in distance on the 6MWT (53 m; an 18% increase from baseline) and physical limitation score from the QoL questionnaire (7 points; a 13% increase from baseline). The increase in distance on the 6MWT mirrors the results obtained in a previous randomized trial: 254 m at baseline, 288 m at 30 days, and 297 m 1 year after TAVI [16].

One explanation for our findings is that daily activity does not actually increase in a relatively older population after TAVI, simply because they do not have to or do not want to increase it (ie, lack of necessity or motivation). In such cases, step count and HR are not expected to increase. However, distance on the 6MWT increased significantly after 1 year in a cohort randomized to TAVI compared to no change in those randomized to medical therapy [17], arguing against a Hawthorne effect. A second explanation for our findings is that QoL questionnaires and the 6MWT [4] generate abundant motivation in the hospital setting, which does not correspond with daily life. In this case, the findings on standard tests (including the 6MWT and QoL questionnaires) would appear to have improved, whereas parameters on the health watch would remain unchanged (eg, the daily total number of steps). Thus, both tests may be accurate given their circumstances. A third explanation is that the health watch parameters contain bias or imprecision, which their paired comparison, even in a reasonably sized cohorts such as our own. This seems to be an interesting topic for further research.

Novel metrics such as the CEES may be valuable parameters in the future or in other settings for evaluating cardiac energy efficiency. The steepness of the slope objectively quantifies the relationship between HR and TEE. A less steep slope corresponds with a lower CEES value. In such cases, more energy is needed to maintain HR than when the slope is steeper (and CEES value higher). In this study, there was a significant increase in the CEES for the good responders cohort, which was accompanied by a significant increase in moderate activity time. There was no significant improvement in the CEES or moderate daily activity time in patients who had no or moderate improvement in distance on the 6MWT (<40 m) after TAVI. This novel metric could be used in future research as a tool to identify patient improvement after a TAVI intervention, independent of subjective variables. More research on this metric is warranted.

How to best identify patients whose symptoms benefit from treatment with TAVI remains an important and unanswered clinical question. A post hoc analysis of a randomized trial of TAVI compared to a surgical aortic valve replacement demonstrated that 36% of patients had no change in 6MWT after outcomes after 30 days and 12 months, and 23%-28% of patients demonstrated no improvement in their QoL questionnaire scores (albeit using a different tool than the one used in our study) [16]. When considering an intervention, both procedural risks and economic costs should be balanced against the potential improvement in QoL. Since the patients who commonly qualify for TAVI treatment are relatively older and frailer, an increase in physical performance can be equally or even more important than an increase in life expectancy. With increasing health care costs, the benefit of an intervention should be clear and personalized [18], and for TAVI, this cost-benefit ratio has been disputed [19]. Unfortunately, the overall findings from this study cannot identify patients using the health watch who would be expected to have an above-average response.

The impact of health watches and other sensor technologies on cardiologic care warrants more research. The Apple Heart study [10] found an irregular cardiac rhythm in only 0.52% of over 400,000 people followed up for 8 months, of whom, only 21% completed further testing, and of them, a 34% minority were ultimately diagnosed with atrial fibrillation, accounting for a paltry yield of 153 people, or much lesser than 0.1% of the total. Findings such as these show the inevitable trade-offs between mass testing and the pretest probability of an actionable diagnosis.

### Limitations

Not all patients had available follow-up data, including 11 patients who died. However, follow-up and health watch data were complete for >80% of patients. The second health watch measurement was performed 3 months after the procedure as that timing fit best with the local follow-up protocol. It might be speculated that not all patients have completely recovered at 3 months already. However, most studies comparing TAVI to surgical aortic valve replacement have reported good functional improvement in the TAVI cohort at 30 days and at 6 months [20] compared to the surgical aortic valve replacement group, with no further improvement in the TAVI cohort in 1 year. This implies that most patients are already at full capacity at 3 months’ follow-up.

### Conclusions

This is the first study that compares pre- and post-TAVI outcomes with extensive, 1-week functional assessment with a sophisticated wearable sensor, the Philips Health Watch. Data from the health watch did not register an increase in activity time, total step count, or other parameters after TAVI, whereas the traditional 6MWT and QoL assessment outcomes improved. Watch-based parameters such as these might be less appropriate for measuring treatment effects in the TAVI population. However, our findings relating to the good responder subpopulation suggest that using data such as the CEES parameter derived from wearable device data, might be useful to objectively identify patient improvement after TAVI intervention. This seems to be interesting for further research.

## Supplementary material

10.2196/53964 Multimedia Appendix 1 Procedural data.

## Abbreviations

6MWT: 6-minute walking test
CEES: cardiac energy expenditure slope
HR: heart rate
MET: Metabolic Equivalent Task
NYHA: New York Heart Association
QoL: quality of life
TAVI: transcatheter aortic valve implantation
TEE: total energy expenditure
