# Supplementary material for: Cardiac Health Assessment Using a Wearable Device Before and After Transcatheter Aortic Valve Implantation: Prospective Study
Source: JMIR Mhealth Uhealth. 2024 Jun 3;12:e53964. doi: 10.2196/53964 (PMC11185971; doi:10.2196/53964)
Supplement: Multimedia Appendix 1 [file mhealth-v12-e53964-s001.docx]

**SUPPLEMENTAL MATERIAL**

**Table S1. Procedural data**

| Characteristic | Total (n=97) | Male (n=56) | Female (n=41) | p value |
| --- | --- | --- | --- | --- |
| TAVI Device | |  |  |  |
| Allegra | 2 (2%) | 1 (2%) | 1 (2%) |  |
| Edwards | 47 (48%) | 31 (55%) | 16 (39%) |  |
| Evolute | 48 (49%) | 24 (43%) | 24 (59%) |  |
| Post-operative | |  |  |  |
| Procedural death | 1 (1%) | 0 (0%) | 1 (2%) | 0.42 |
| LBBB | 22 (23%) | 14 (25%) | 8 (20%) | 0.50 |
| Pacemaker implantation | 10 (10%) | 6 (11%) | 4 (10%) | 0.86 |
| Major bleeding | 15 (15%) | 10 (18%) | 5 (12%) | 0.45 |
| CVA | 1 (1%) | 0 | 1 (2%) | 0.43 |
| Coronary occlusion | 1 (1%) | 0 | 1 (2%) | 0.43 |

Abbreviations: TAVI = transcatheter aortic valve implantation; LBBB = left bundle branch block; CVA = cerebrovascular accident. Summary values represent number (%).

**Table S2. Univariate analysis of good responders watch data pre-TAVI**

|  | β | S.E. | p value |
| --- | --- | --- | --- |
| Resting heart rate (1/min) | 0.02 | 0.03 | 0.46 |
| Respiratory rate at rest (1/min) | 0.07 | 0.13 | 0.58 |
| Heart rate (1/min) | 0.02 | 0.03 | 0.52 |
| Heart rate during sleep (1/min) | 0.02 | 0.03 | 0.41 |
| Respiratory rate during sleep (1/min) | 0.10 | 0.12 | 0.41 |
| Daily percentage of HR observations < 60. bradycardia | 0.00 | 0.01 | 0.78 |
| Daily percentage of HR observations > 100. tachycardia | 0.06 | 0.06 | 0.33 |
| Daily total number of steps | 0.00 | 0.00 | 0.78 |
| Daily cumulative active energy expenditure (kcal) | 0.00 | 0.00 | 0.98 |
| Daily cumulative total energy expenditure (kcal) | 0.00 | 0.00 | 0.64 |
| Slope of log(HR/TEE) | -3.92 | 2.59 | 0.13 |
| Daily sleep time (hours) | -0.13 | 0.15 | 0.37 |
| Daily basal activity time (min) | 0.00 | 0.00 | 0.46 |
| Daily light activity time (min) | 0.00 | 0.00 | 0.24 |
| Daily moderate activity time (min) | 0.00 | 0.00 | 0.53 |
| Daily high activity time (min) | -0.04 | 0.07 | 0.61 |
| Daily total active (min) | 0.00 | 0.00 | 0.78 |

Abbreviations: β = unstandardized beta. S.E. = standard error for the unstandardized beta.

**Table S3. Good responders watch data pre versus post TAVI**

|  | Good responders pre TAVI (n=43) | Good responders post TAVI (n=40) | p value |
| --- | --- | --- | --- |
| Resting heart rate | 62.6 ± 10.3 | 62.3 ± 7.9 | 0.90 |
| Respiration rate at rest | 16.5 ± 2 | 16.2 ± 1.9 | 0.70 |
| Heart rate | 70.2 ± 9.6 | 70.2 ± 7 | 0.95 |
| Heart rate during sleep | 64.3 ± 10 | 63.4 ± 7.8 | 0.61 |
| Respiration rate during sleep | 16.1 ± 2.3 | 15.8 ± 2.3 | 0.67 |
| Daily percentage of HR observations < 60, bradycardia | 13.3 [0.8-3.0] | 15.0 [3.5-35.5] | 0.46 |
| Daily percentage of HR observations > 100, tachycardia | 1 [0.3-1.0] | 1.6 [0.7-2.7] | 0.86 |
| Daily total number of steps | 3633 [2763.0-5135.0] | 4488.5 [2965.5-6664.5] | 0.18 |
| Daily cumulative active energy expenditure (kcal) | 733 ± 221.4 | 790.6 ± 218.4 | 0.15 |
| Daily cumulative total energy expenditure (kcal) | 2310.7 ± 428.1 | 2372.8 ± 466.1 | 0.19 |
| Slope of log(HR/TEE) | 0.26 ± 0.10 | 0.27 ± 0.09 | 0.04 |
| Daily sleep time (hrs) | 8.2 [7.0-8.7] | 8.3 [7.3-9.2] | 0.28 |
| Daily basal activity time (mns) | 209.0 [173.4-261.3] | 200.1 [166.2-264.6] | 0.94 |
| Daily light activity time (mns) | 195.2 ± 88.8 | 204.9 ± 77.1 | 0.63 |
| Daily moderate activity time (mns) | 20.2 [8.8-66.3] | 66.8 [29.5-113.4] | 0.01 |
| Daily high activity time (mns) | 0.0 [0.0-0.0] | 0 [0.0-2.0] | 0.19 |

**Table S4. Correlations from Health watch parameters, 6MWT, and questionnaire**

| ** Correlation is significant at the 0.01 level (2-tailed). |
| --- |
| * Correlation is significant at the 0.05 level (2-tailed). |
| * pre = pre TAVI; post = post TAVI |

**Figure S1. Scatter plot of Health watch parameters, 6MWT, and questionnaire**

**
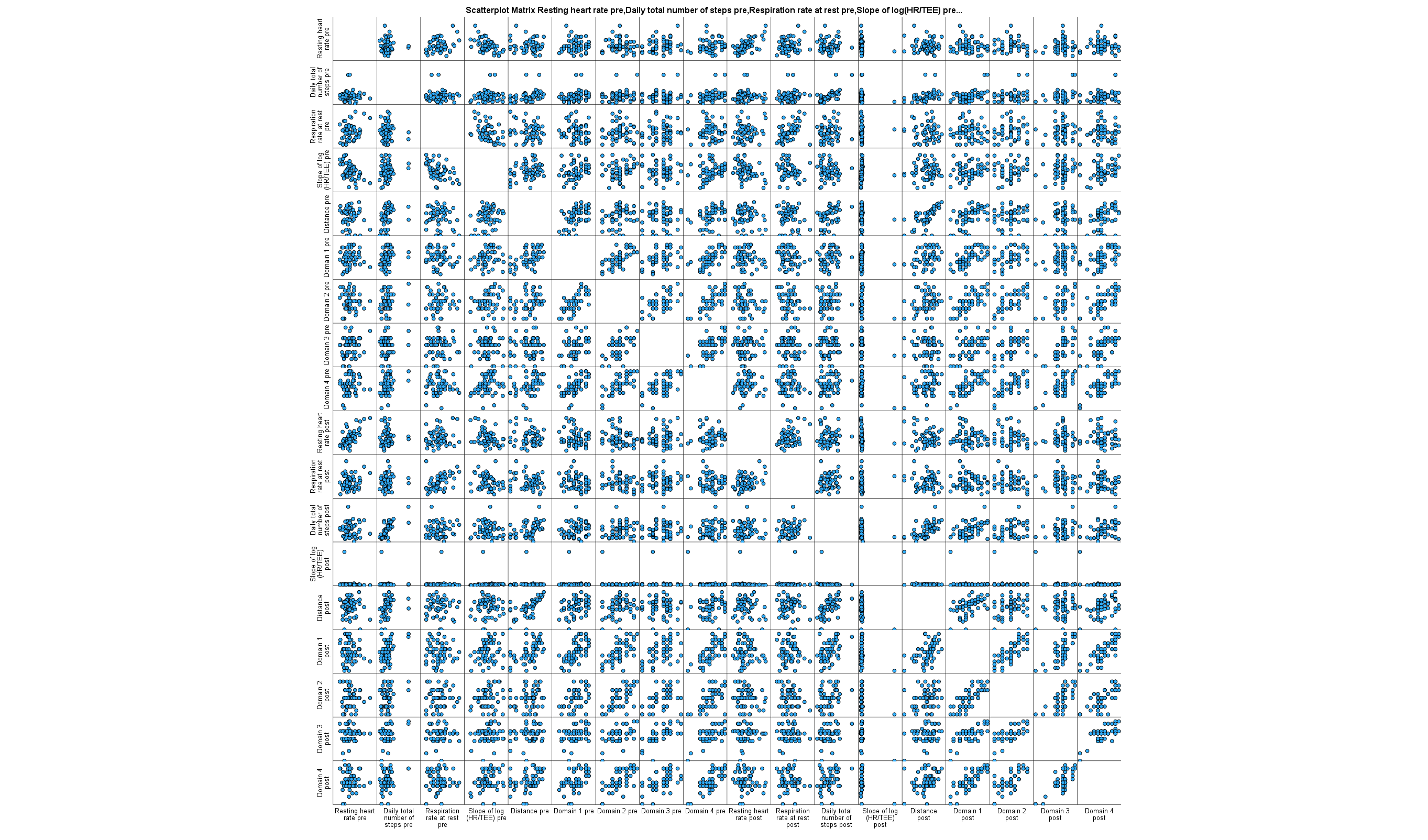
**
